# Unveiling a Critical Subacute Complication: Right Apical Septum Pacemaker Lead Dislodgement Leading to Right Ventricular Perforation and Minimal Pericardial Effusion

**DOI:** 10.7759/cureus.57641

**Published:** 2024-04-05

**Authors:** Wassim Abouzeid, Ahmad W Haddad, Noreen Mirza, Eyad Ahmed, Joaquim Correia, Addi Suleiman

**Affiliations:** 1 Internal Medicine, Saint Michael's Medical Center, Newark, USA; 2 Medicine, Saint Michael's Medical Center, Newark, USA; 3 Cardiology, Saint Michael's Medical Center, Newark, USA

**Keywords:** sick sinus syndrome, pacemaker lead complication, right ventricular perforation, pericardial effusion, pacemaker complication

## Abstract

The implantation of cardiac pacing devices, such as pacemakers and implanted cardioverter-defibrillators (ICDs), has significantly improved patient outcomes in the treatment of a range of cardiac arrhythmias. Right ventricular (RV) pacing lead perforation is an uncommon but potentially dangerous complication that can occur despite technical breakthroughs. RV lead perforation, which can result in right ventricular perforation as well as possible pulmonary or vascular harm, is caused by the pacing lead breaking through the myocardial wall. Despite being rare, this complication warrants attention because of the risks for morbidity and mortality that are involved. We present a case of right ventricular perforation caused by a pacemaker lead and examine the nuances of RV lead perforation in this instance, including its prevalence, clinical presentation, diagnostic difficulties, and treatment strategies, illuminating the many factors to be taken into account while properly managing this issue.

## Introduction

Following pacemaker implantation, acute problems such as lead dislodgement, pneumothorax, and cardiac perforation are uncommon but serious. The lead can pierce through the heart and enter the epicardial space, pericardium, or chest wall. Lead perforation can occur early or late [1]. Sometimes, these perforations are clinically occult, meaning they do not cause symptoms like discomfort or pericardial effusion [1]. Despite its limitations in differentiating between the ventricular chamber, myocardial, and pericardium, a chest X-ray taken from two distinct perspectives can help demonstrate perforation. For lead tip identification, a cardiac computed tomography (CT) scan is more trustworthy. Repositioning the leads to the desired position frequently involves a risk of pericardial effusion, infection, and extended hospital stay [2]. We present the case of a 77-year-old man who had his pacemaker leads removed after they were punctured into the wall of his right ventricle.

## Case presentation

**Figure 1:**
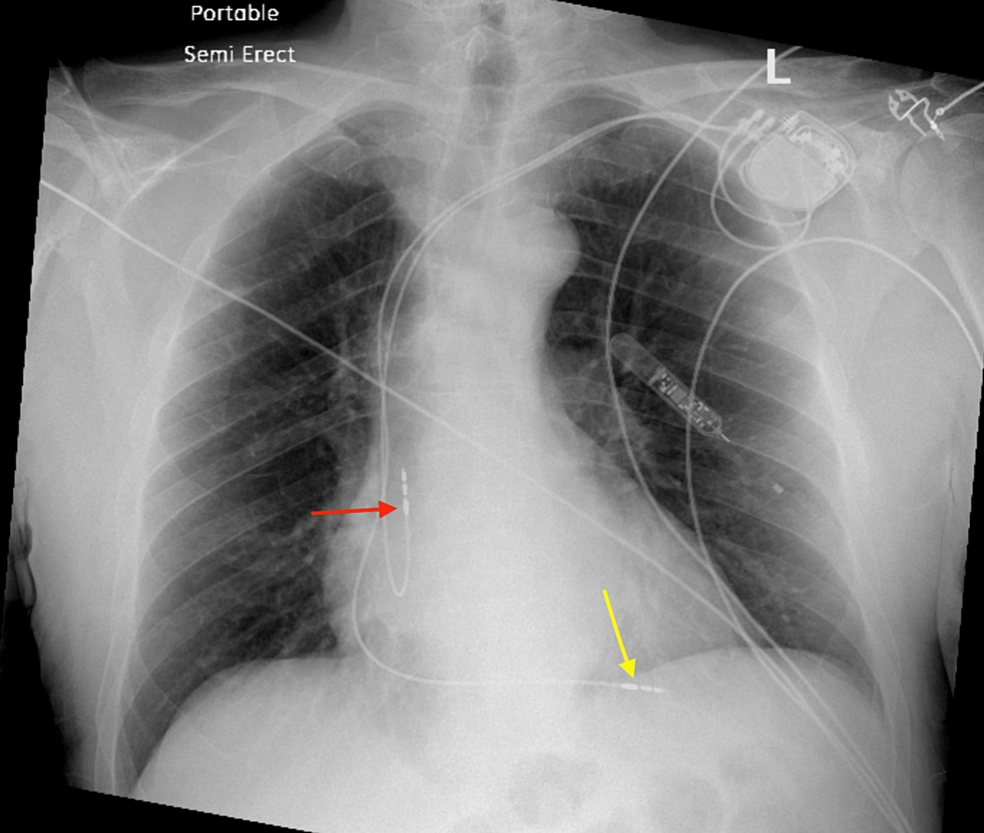
Chest radiograph (CXR) PA view Chest X-ray PA view after pacemaker insertion shows pacemaker leads in the right atrium (red arrow) and right ventricle (yellow Arrow).

A 77-year-old male with a past medical history of hypertension, paroxysmal atrial fibrillation, sick sinus syndrome, and hypothyroidism was admitted to the hospital for elective permanent pacemaker insertion due to documented symptomatic pauses and paroxysmal atrial fibrillation on a loop recorder that was inserted after syncopal episodes in the past. A BIOTRONIK dual chamber pacemaker device was successfully inserted and the RV lead was screwed in at the apical septum part of the right ventricle without complication. The pacemaker device was set to DDD mode with heart rate limits set between 70 and 130 beats per minute (bpm), with an output of 3.5 V in both chambers. Follow-up chest radiograph (CXR) confirmed lead placement in both the right atrium (RA) and right ventricle (RV) as shown in Figure 1. A follow-up electrocardiograph (ECG) was done and atrial-ventricular paced rhythm was appreciated with a heart rate of 90 beats per minute. The patient was discharged home on apixaban 5 mg.

**Figure 2:**
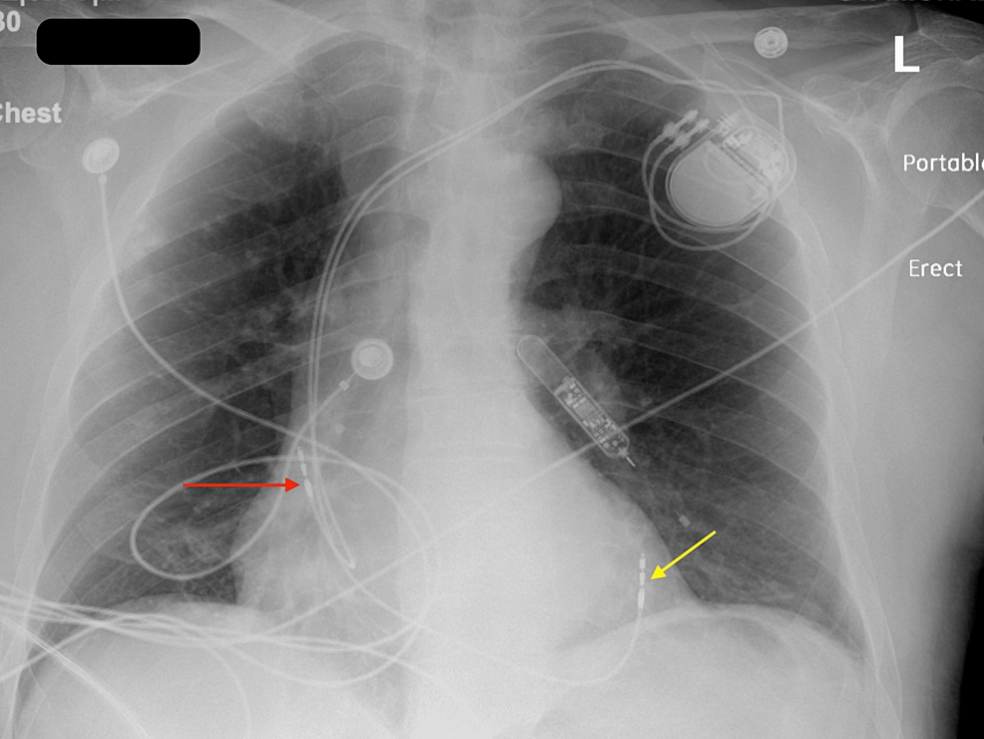
Chest X-ray PA view on the presentation Chest X-ray PA view on presentation shows the right atrial pacemaker lead in place (red arrow), and dislodgement and migration of the right ventricular pacemaker lead (yellow arrow).

**Figure 3:**
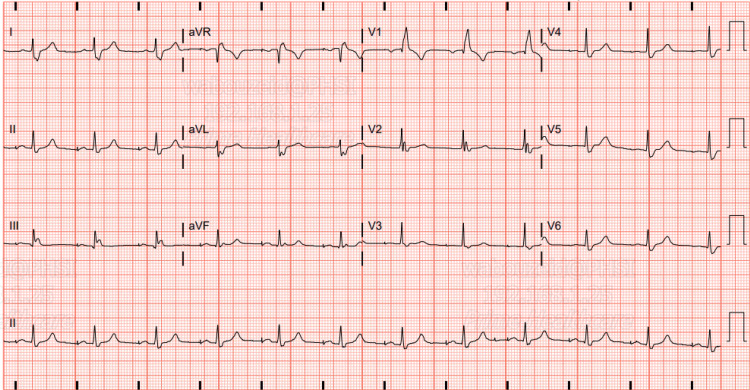
ECG on presentation ECG on presentation after switching pacemaker mode to AAI shows atrial paced rhythm

**Figure 4:**
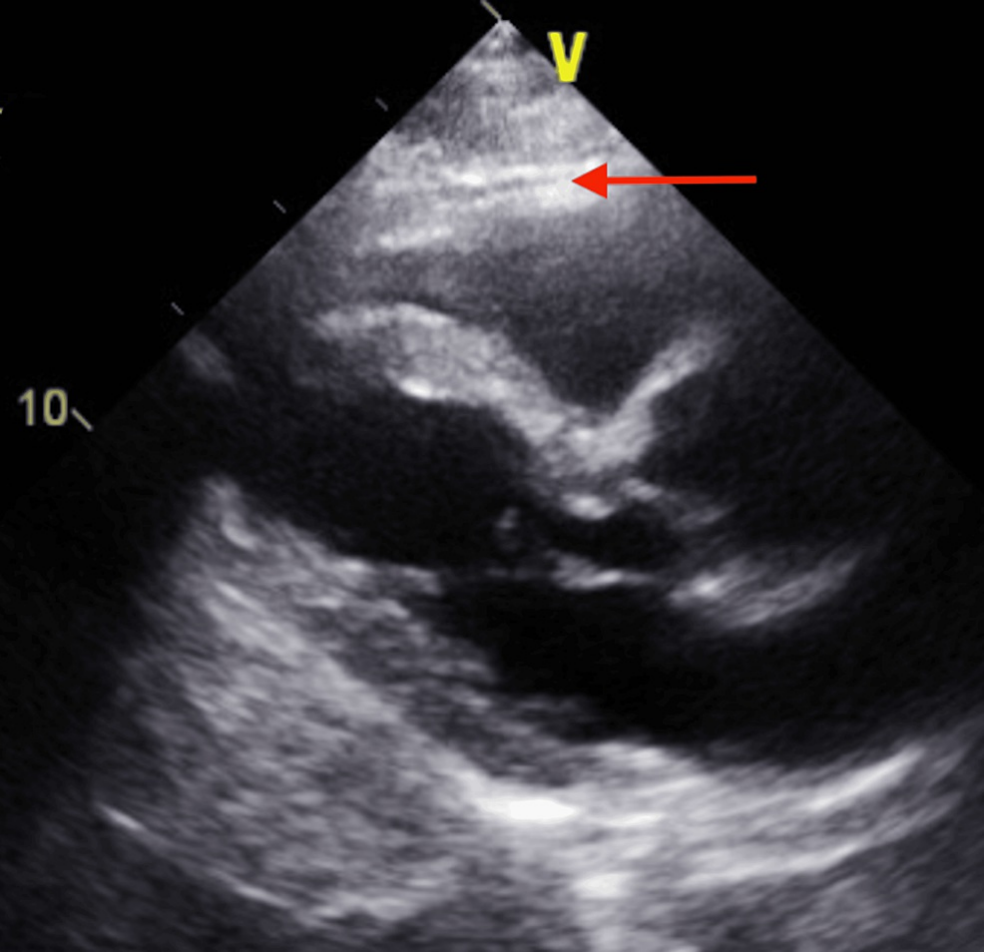
Transthoracic echocardiogram (TTE) on admission. TTE on admission long axis parasternal view shows minimal pericardial effusion (red arrow).

**Figure 5:**
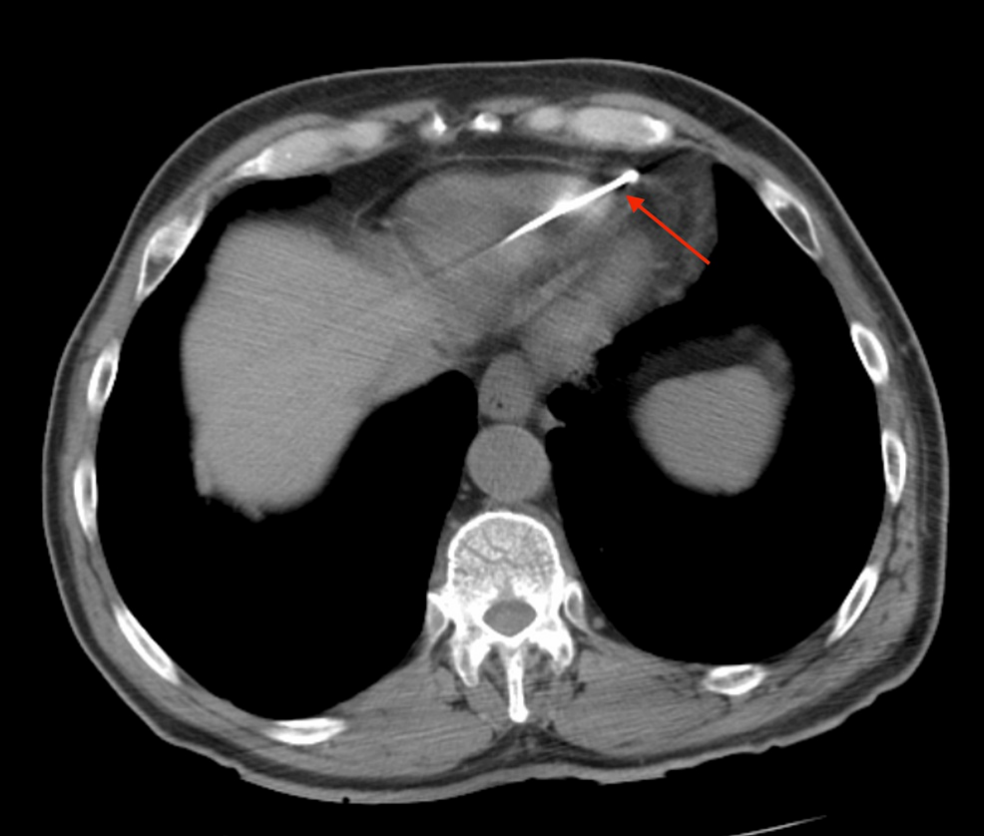
CT chest on presentation CT chest on presentation shows the right ventricular pacemaker lead perforating the right ventricle and the tip of the lead in the pericardium (red arrow).

A week after discharge, the patient presented to the ED complaining of mild left-sided pleuritic chest pain. Upon evaluation, the patient blood pressure was stable along with other vital signs. Chest radiograph (CXR) showed a dislodged RV lead and movement toward the cardiac apex (Figure 2). Troponin and BNP remained within normal limits and haemoglobin levels were at 14 g/dL. Dur to the presence of normal atrioventricular nodal conduction, the device mode was changed to AAI, The patient reported immediate relief in his chest pain, and a confirmatory ECG showed atrial paced rhythm with a heart rate of 70 bpm (Figure 3). Transthoracic echocardiography (TTE) showed minimal anteriorly located pericardial effusion (Figure 4). A confirmatory computed tomography (CT) scan of the chest showed RV perforation and the presence of the RV pacemaker lead within the pericardium (Figure 5).

**Figure 6:**
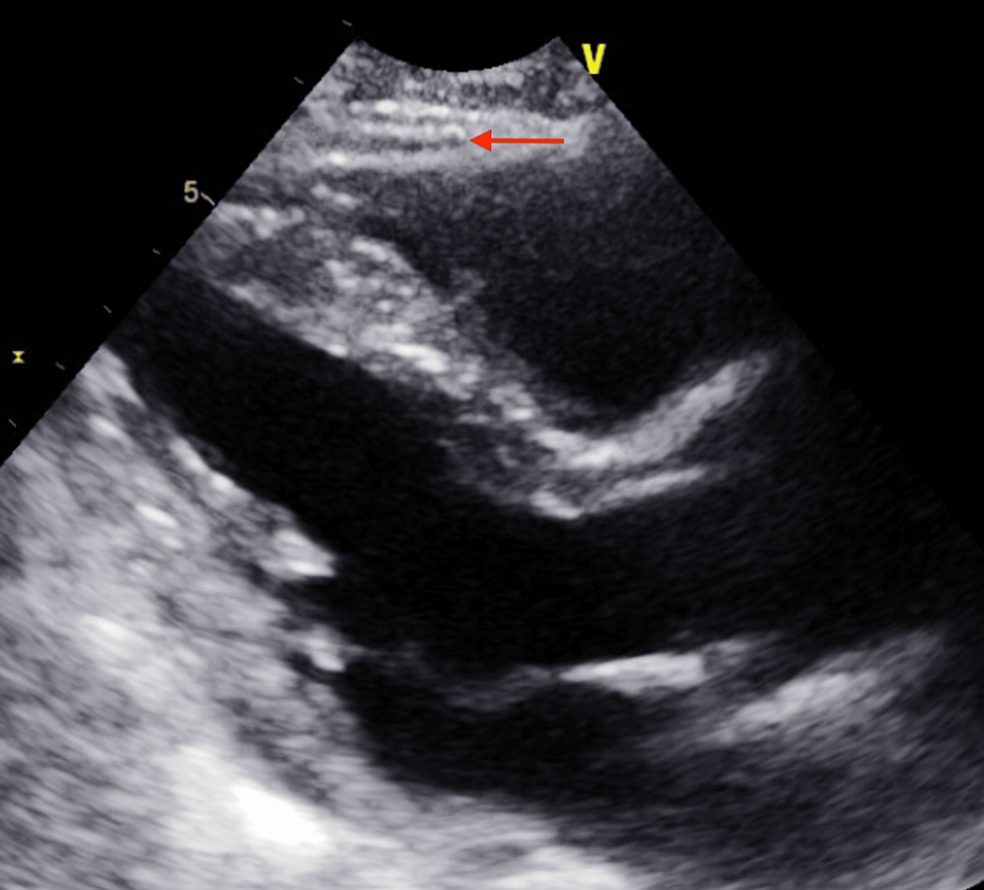
Transthoracic echocardiogram (TTE) after lead removal Transthoracic echocardiogram (TTE) long axis parasternal view after removal of the right ventricular lead shows minimal pericardial effusion and about the same amount prior to lead removal.

**Figure 7:**
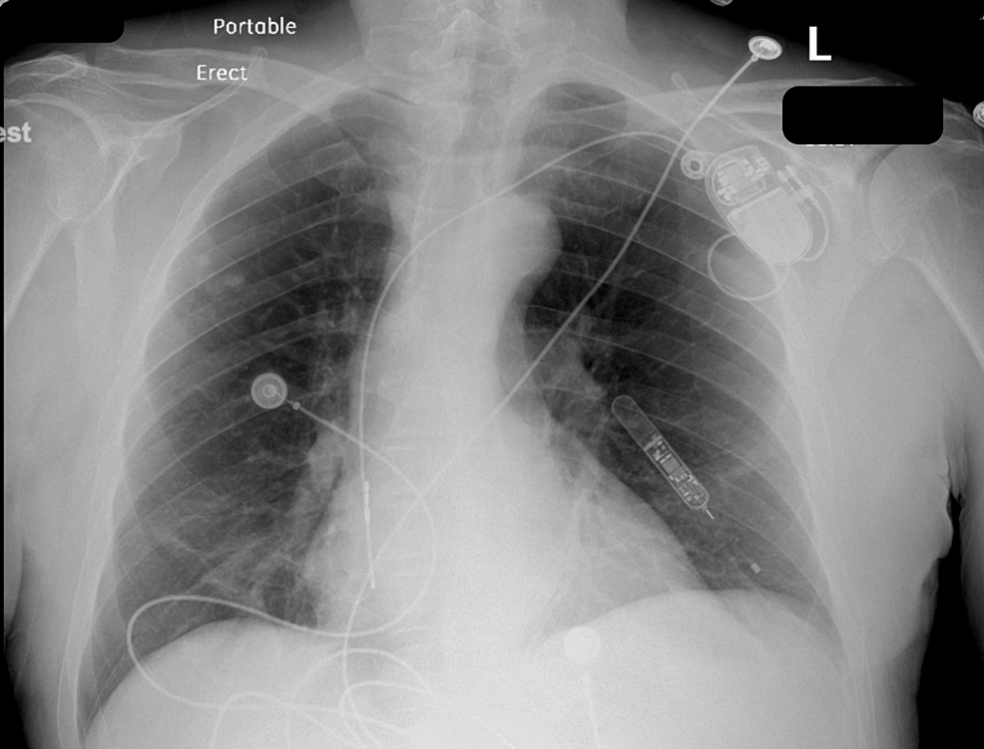
Chest X-ray after Right Ventricular lead removal Chest X-ray PA view shows the right atrial pacemaker lead in place.

As the patient remained hemodynamically stable, the plan was made to remove the RV lead in the operating room under transesophageal echocardiography and Cardiothoracic surgery on backup. The RV lead retrieval was achieved with no immediate postoperative complication, and a TTE post-RV lead removal showed no change in the previously noted minimal pericardial effusion (Figure 6); the CXR showed normal cardiac silhouette and no evidence of increase in pericardial effusion (Figure 7). The patient was monitored for two days after the procedure. He was hemodynamically stable throughout hospital admission and reported resolving his left-sided chest pain. Subsequently, the patient was discharged home.

## Discussion

Rarely when an RV pacing lead to moves outside of the pericardium, it can result in pulmonary or vascular injury and even right ventricular perforation. Although this is a rare occurrence, ventricular perforations can still happen, with some reported cases starting within twenty-four hours after the device is placed [1]. However, the occurrence of this complication might be overestimated in many studies, as it may not cause any symptoms in some people. In the OPTIMUS registry, which tracks the long-term performance of Saint Jude Medical leads, RV perforation was noted in 0.5% of permanent pacemaker (PPM) leads and 0.33% of implantable cardioverter defibrillator (ICD) leads [2].

Patients with late-stage RV lead perforation may present with shortness of breath and sharp chest discomfort; severe pericardial effusion and tamponade are less common [3]. This could be due to the lead itself, fibrosis, or the ventricular wall's self-sealing characteristics [3]. In addition, pacing or sensing malfunctions are frequently reported. Other issues that have been seen in the past include chest muscle twitching [4], hiccups [5], chest wall hematoma [6], rib perforation [7], pneumonia, and hemothorax [8]. In some cases, lead migration can result in perforation of the left liver lobe or migration into the anterior left pleural space [8,9].

Increasing the force delivered to an area can increase the risk of perforation, especially if the lead's design features a stiff tip with a smaller diameter and a lead body construction that creates forward pressure on the lead during an extra loop. However, newer leads have softer and more flexible tips that reduce stress on the wall, thereby reducing the rate of perforation [10]. According to certain studies, other factors have been identified as contributing to the problem at hand. These factors include low body weight, advanced age, female gender, and the use of anticoagulants or steroidal drugs within the first seven days post-implantation [11].

Lower rates of cardiac perforation are linked to high right ventricular systolic pressure (>35 mmHg), which may be because of concomitant RV hypertrophy [11]. The myocardium, the heart's thin muscle layer, can render the heart vulnerable to puncture. According to a study conducted by Akyol et al., implanting a pacemaker lead in a patient with dilated cardiomyopathy and myotonic muscular dystrophy resulted in perforation of the right ventricle [12]. It is essential to note that blunt chest trauma can significantly elevate the risk of heart perforation, especially in the early post-implantation period [13].

The majority of late perforations don't show any symptoms, and suspicion is aroused when regular analysis reveals device dysfunction. It is possible to confirm a diagnosis of ventricular lead perforation using echocardiography, fluoroscopy, chest CT scan, or chest radiography.

The problem can be readily identified by fluoroscopy and chest radiography when the lead migrates too far away from the heart. However, these tests are frequently non-diagnostic in situations where there is only a little cardiac perforation. When pacing wire perforation is detected, and the wire's course can be seen in the spatial orientation of the echocardiography beam, two- and three-dimensional echocardiography can also be useful [14]. Following implantation, echocardiography-detected pericardial effusion may indicate a lead perforation; however, other mechanisms, such as immune-mediated mechanisms or traumatic inflammation of the myocardium and pericardium from the lead screw, can also induce pericardial effusion [15].

It is still uncertain how best to handle lead perforations. Asymptomatic lead perforation with otherwise normal function is very common. Based on our experience and the opinions of certain specialists, it is not necessary to extract an asymptomatic, accidentally found, chronically perforated lead [15]. However, some clinicians would recommend extraction of these leads [15]. Patients who experience symptoms or device malfunctions, on the other hand, will need the necessary adjustments to address their issues. Although medication is rarely necessary for hemodynamically stable patients with postimplantation pericardial effusion and normal device function, they should be constantly monitored because of the possibility of cardiac tamponade [16]. Sometimes, reprogramming of the device is sufficient to correct the situation [17].

Once the active fixation screw has been retracted, the lead can be manually withdrawn by direct traction with the use of a standard stylet in the event of a symptomatic heart perforation or lead malfunction without a considerable pericardial effusion. A cardiac surgical team should be available for this treatment, which should be carried out in a hybrid operating room under TEE supervision. It is possible to move the leads to a different location [3].

## Conclusions

A rare but dangerous side effect of PPM and ICD installation is late RV perforation. It could show up as a potentially fatal illness like tamponade or be totally asymptomatic. Heart CT with ECG gating and no contrast is among the best diagnostic techniques. The topic of optimal management still needs to be determined. It is not necessary to treat a continuously perforating lead that was accidentally discovered. It is usually safe to remove the lead in a hybrid operating room when the patient has symptoms or when there is lead malfunction. However, surgery can be required if there are additional visceral injuries.
